# The Impact of COVID-19 on Patients With Obsessive-Compulsive Disorder in Gulf Countries: A Narrative Review

**DOI:** 10.7759/cureus.67381

**Published:** 2024-08-21

**Authors:** Jihad Algadeeb, Mohammed J Alramdan, Rahma B AlGadeeb, Kumail Naser Almusawi

**Affiliations:** 1 Preventive Medicine Department, Al-Ahsa Health Cluster, Ministry of Health, Al-Ahsa, SAU; 2 Community Wellness Department, Al-Ahsa Health Cluster, Ministry of Health, Al-Ahsa, SAU; 3 Pediatrics Department, King Faisal General Hospital, Al-Ahsa Health Cluster, Ministry of Health, Al-Ahsa, SAU

**Keywords:** epidemiology, mental health, obsessive-compulsive disorder, covid-19, gulf region

## Abstract

Gulf countries, like other parts of the world, were affected by the coronavirus disease 2019 (COVID-19) pandemic. Along with its biological effects, the pandemic has had serious psychological and social effects. The pandemic-associated general stress and the increased efforts of handwashing and general hygiene might trigger obsessive-compulsive disorder (OCD). The objective of this narrative review was to explore the influence of the COVID-19 pandemic on the prevalence, severity of symptoms, and accessibility of treatment for OCD in Gulf countries. A comprehensive literature search was conducted to review and collect research and/or reports on the prevalence, symptoms, diagnosis, and treatment adaptations and strategies of OCD during the ongoing COVID-19 pandemic in seven Arab Gulf countries. The search spanned from the onset of the crisis in 2020 to 2024. Peer-reviewed articles and reports were sourced from PubMed/Medline and Google Scholar, while abstracts presented at the American Society of Clinical Oncology (ASCO) and the European Society for Medical Oncology (ESMO) congresses were also included in the review. A total of four studies from Saudi Arabia, two from the United Arab Emirates, and two from Qatar were retrieved for analysis. These studies focused on investigating the impact of the pandemic on OCD. Studies from Kuwait, Oman, and Bahrain reported a negative impact of the pandemic on mental health, yet no specific data were provided. The studies highlighted an increased prevalence of OCD symptoms, both in terms of the incidence of new symptoms and the exacerbation of existing pre-pandemic manifestations. Furthermore, individuals with pre-existing psychological disorders or OCD were particularly susceptible to the negative impact of the pandemic. A review of local studies and reports from the Arab Gulf region reveals a striking paucity of research on the impact of the COVID-19 pandemic on OCD. The pandemic has been associated with an increase in the prevalence of OCD, the onset of new symptoms, and the worsening and exacerbation of existing pre-pandemic obsessions and compulsions.

## Introduction and background

Obsessive-compulsive disorder (OCD) is a common mental disorder characterized by undesirable and distressing obsessions and/or compulsions in the form of repetitive thoughts or behaviors. OCD becomes a focus of interest because it is associated with major impairment in social functioning and quality of life, decreases academic performance and work productivity, and disturbs family relationships. Thereby, it constitutes a great burden with increased healthcare and economic expenditures [1]. The early age of onset, diverse symptoms, chronic course, and frequent comorbidity with other psychiatric disorders complicate the disorder, with a high rate of underdiagnoses and undertreatment [2].

OCD is among the 10 most disabling medical conditions in the world, and it is also the fourth most common mental disorder [3]. Globally, the estimated lifetime prevalence of OCD in the general population ranges from 1% to 3.2%. Also, the 12-month and 30-day prevalence estimates are in the range of 0.3-3.0% and 0.3-3.1%, respectively [4]. Similar figures have been documented in the Middle East and North Africa [5] and the Eastern Mediterranean Regions [6,7]. Furthermore, Fawcett et al. [8] concluded that females and younger adults are at greater risk of experiencing OCD in their lifetime than their counterparts.

The coronavirus disease 2019 (COVID-19) pandemic is the biggest health challenge faced by the world in recent history. It affected most countries with millions of deaths worldwide due to infection and its complications [9]. The Gulf countries are a unique group of countries located adjacent to the Arabian Gulf, including Bahrain, Kuwait, Oman, Qatar, Saudi Arabia, and the United Arab Emirates (UAE). They are characterized by social differences from Western differences, a young population structure, a significant proportion of foreign workers, and a high level of shared religious observance [10].

Like other parts of the world, Gulf countries were also affected by the COVID-19 pandemic. The incidence of the COVID-19 virus infection initially grew slowly in each of the seven Gulf countries, and then confirmed cases of COVID-19 continued to grow rapidly. The Gulf’s large guest worker population created an environment that was encouraging the rapid spread of the virus. The total number of cases in the Gulf reached over 1 million in early January 2021 and 2 million by the beginning of June as the Gulf experienced multiple waves of the virus. However, after the emergence of the Omicron variant by mid-January 2022, cases in the Gulf spiked again. The region had seen over 3.7 million cases by mid-June 2022 [10]. As soon as the cases of COVID-19 were confirmed, the Gulf countries’ governments initiated restrictive measures to slow the spread of infection.

Along with its biological effects, the COVID-19 pandemic has serious psychological and social effects. The COVID-19 pandemic and its associated restrictions may contribute to a deterioration in mental health and are expected to have a negative effect on OCD patients [11]. The pandemic-associated general stress on health and community and the increased efforts of handwashing and general hygiene as an essential step in COVID-19 prevention might trigger the obsession with contamination and compulsive washing of hands [12]. In this context, Chen et al. demonstrated that COVID-19 influenced symptoms of mental disorders [13]. Regionally, in the Gulf Cooperation Council countries, Al-Mutawa and Al-Mutairi [14] reported an increased risk of mental health problems among the general population during the COVID-19 pandemic.

Alternatively, some researchers thought that individuals with OCD tend to direct their attention toward their inner thoughts [15] and thus might be less affected by external events, and the presence of a new virus would not necessarily increase the fear of contamination. Furthermore, these patients might instead feel more protected during the quarantine. They also might feel some relief because their concerns surrounding cleanliness are justified [16].

This narrative review aimed to provide a comprehensive account of the impact of the COVID-19 pandemic on the prevalence, severity of symptoms, and access to treatment of OCD in Gulf countries. Additionally, it sought to identify any changes in therapeutic approaches to OCD during the pandemic.

## Review

Methods

Search Strategy

A comprehensive literature search was conducted to collate research and reports on the prevalence, symptoms, diagnosis, and treatment adaptations and strategies associated with OCD during the ongoing global pandemic. The search spanned the seven Arab Gulf countries from the onset of the crisis in 2020 until May 31, 2024. A review of the literature was conducted using PubMed/Medline and Google Scholar for peer-reviewed articles and reports, and the ASCO and ESMO congresses were searched for relevant abstracts presented during the studied time frame.

We performed a comprehensive literature review of reports of OCD in Arab Gulf countries by using the following keywords (“obsessive-compulsive disorder” OR “psychological distress” OR “mental disorder) AND (“epidemiology” OR “incidence” OR “prevalence”) AND (“Gulf region” OR “Gulf countries” OR “Gulf nations” OR “Gulf populations” OR “Saudi Arabia” OR “ Qatar” OR “Oman” OR “ Bahrain” OR “ Kuwait” OR “ United Arab Emirates”) AND (“SARS-CoV-2” OR “ COVID-19”) AND (“new symptoms”) AND (“severity” OR “exacerbations”) AND (“treatment” OR “therapy” OR “adaptation”).

Inclusion and Exclusion Criteria of the Studies

All national and international studies and reports published in English within the time frame on the prevalence, diagnosis, severity, and treatment of OCD in the Gulf Arab countries were included. We excluded non-English publications, studies conducted outside the specified time frame of 2020 to May 31, 2024, studies that did not focus on the Arabian Gulf countries, as well as case studies, case series, clinical commentaries, and review articles. These exclusion criteria were applied to ensure the inclusion of studies with stronger evidence and direct relevance to the research objectives. The final number of included studies for this review was eight studies.

Main findings

Impact of the COVID-19 Pandemic on Prevalence and Symptoms Severity of OCD

In Saudi Arabia, according to the Saudi National Mental Health Survey, it is estimated that 2-4% of individuals in the general population will develop OCD before the age of 18 years, and the lifetime prevalence of OCD is 4.2%. The OCD symptoms follow a chronic course, with exacerbations accompanying periods of life stress [17]. Studies during the COVID-19 pandemic reported an increased prevalence of OCD symptoms, both in the form of incidence of new symptoms and exacerbations of already existing ones. A survey study done during the initial stage of the pandemic in Saudi Arabia showed an increased prevalence of OCD symptoms; 62.4% of the participants most likely had OCD. The study also conveyed a high prevalence among males, the older population, and those with a prior COVID-19-positive diagnosis [18]. Further, in July 2020, a cross-sectional survey of 2909 participants in Saudi Arabia revealed a prevalence of new-onset obsessions and compulsions of 58% and 46%, respectively. In addition, participants with new-onset obsessions and compulsions reported significantly high stress levels (57.5% and 51.4%) [19]. During September and November 2020, another study screened the 1,190 general populations who had COVID-19 infection or were in contact with a COVID-19 patient through an online survey for OCD symptoms. New OCD symptoms were reported in a high proportion of the participants (82%). Positive OCD screening was significantly higher in participants who reported previous psychological illness (87.6% vs. 80.9%), those who followed news related to COVID-19 daily (88.7% vs. 76.1%), and participants who had not acquired the infection (82.9%) compared to those who were infected with COVID-19 (72.3%) [20]. A more recent study, between July and October 2021, evaluated 356 adult patients who had survived a previous COVID-19 infection for OCD symptoms. The study reported an overall prevalence of OCD symptoms of 33%, with an increased prevalence of new OCD symptoms, especially the purity and cleanliness compulsions. As well, the recorded OCD symptoms were higher among psychiatric and OCD patients [21]. The detected prevalence (33%) at the end of 2021 is still higher than the prevalence of OCD symptoms reported before the COVID-19 pandemic among the general population [17], and college or secondary school students in Saudi Arabia (20%-23%) [22,23] but it is considered lower than the aforementioned prevalence reported during the initial waves of the pandemic during the year 2020 [18-20].

Similarly, studies from the UAE suggested an increased prevalence of OCD during the COVID-19 pandemic. Al Hassani and Mufaddel [24] reported a 31% prevalence rate of mild to severe degrees of OCD symptoms among 702 residents during an earlier stage of the pandemic (between October 2020 and January 2021). They also showed an increased prevalence of OCD among people with positive COVID-19 infection. Another study in the UAE during the year 2021 assessed the severity of obsessions and compulsions among 343 adult residents with no prior diagnosis of OCD through an online survey. The study revealed an overall prevalence of about 33%. Furthermore, one-third of the female and one-fourth of the male participants recorded different grades of OCD-like symptoms [25].

In Qatar, Khan et al. [26] reported significant pandemic fears associated with obsessive-compulsive symptoms and explored the association of the pandemic with obsessive-compulsive symptoms in adolescents with preexisting mental and behavioral disorders. Moreover, a telephonic questionnaire-based study enrolled adults with pre-existing OCD specifically with major symptoms of fear of contamination and washing compulsions, which revealed increased severity of OCD symptoms only among those with the duration of OCD diagnosis of less than 10 years [27]. Another study in Qatar revealed an increased prevalence of anxiety symptoms, mainly OCD symptoms, among children presenting to emergency care during the pandemic [28].

An online survey including the Brief Symptom Inventory (BSI) scale was used to collect data from the 380 students of Kuwait University after the COVID-19 pandemic between September and December 2021. The study documented a 17% and 43% prevalence of high and moderate levels of mental disorders, and OCD was the most common one [29].

The COVID-19 epidemic has had a severe impact on the general population’s psychological health in Oman. There was an increased prevalence of depressive and anxiety symptoms (4.2% and 14.9%) among general populations in one study [30]. Another study confirmed the high rates of depression, anxiety, and stress among medical students during the COVID-19 pandemic [31]. To date, there has been no data published on OCD prevalence during the COVID-19 pandemic in Qatar Oman, or Bahrain.

Contributing Factors

The sudden and extreme change in an individual’s daily life during the COVID-19 pandemic translated to severe social and psychological disruptions [32]. The pandemic itself, the governmental measures like lockdowns, the pandemic-related increased deaths, and the lack of effective treatment have led to psychological distress, with reports of moderate to severe increases in anxiety, depression, and stress [33]. OCD patients might be especially in danger of increased psychological distress because many aspects of the COVID-19 disaster focus on uncertainty and cleanliness, which are principal symptoms of many OCD patients. Furthermore, it is known that stress triggers the onset of OCD. In addition, patients with OCD perceive stress higher than subjects without this disorder [34]. The most common OCD symptoms are an obsession with contamination and the compulsion to clean [35]. Since the strategies that combat infectious diseases contain repetitive behaviors by nature, these strategies can worsen OCD [36].

Previous research has shown that COVID-19 patients experience significant psychological distress once they are informed of a positive COVID-19 diagnosis [37]. Lockdowns were also associated with an immediate negative impact on the mental health of general populations [38]. Menon et al. [39] have demonstrated that loneliness is one of the main consequences of COVID-19 pandemics that make people more vulnerable to anxiety and depression.

It is worth mentioning that responses to these circumstances and stresses vary among individuals, and these responses are related to internal factors, such as biological stress response, cognitive capacity, and personality traits, or external factors, such as financial stability and social status [40]. Although the Arab Gulf region showed increased acceptance of mental health disorders due to awareness campaigns and public health education, it is still highly influenced by sociocultural challenges including financial barriers, shame, and stigma [41]. The Gulf society has shown some factors which may also affect individual stress response. The disturbed traditional social patterns due to the rapid growth in population and the process of urbanization, in addition to the cultural and religious factors of the region, could influence the manifestations, clinical course, severity, and treatment response in patients having OCD [42,43].

Access to Treatment

In the era of the COVID-19 pandemic, people having mental health problems faced many barriers to accessing mental health services such as healthcare disruptions, closures, lockdown policies, fear of getting an infection while visiting a care facility, and suspension and cancellation of elective services. In addition, workforce shortages and increased demand for services worsened the condition [44].

Worldwide, some policies and strategies were implemented to address access challenges, such as the growth of telehealth, the expansion of school-based mental health services, and the rollout of hotlines. In the Arab Gulf countries, the most accessible mental health services are psychotherapy and cognitive behavioral therapy, telepsychiatry, and mobile health services, especially during the COVID-19 pandemic [45].

Telepsychiatry refers to looking after patients through telecommunication, mainly via a video or an audio call. Telepsychiatry services were available to varying extents in Qatar, Oman, Saudi Arabia, and UAE before and during the COVID-19 pandemic, and it was effective in improving COVID-19-associated anxiety and depression-related symptoms compared to self-help or internet-based therapy [46].

The delivery of mental health services via telehealth developed sharply during the pandemic, which has helped maintain and expand access to behavioral services during the pandemic [47]. It has been reported that more patients in the UAE used telemedicine during the COVID-19 pandemic for acute, chronic, or COVID-19-related consultations [48]. A study from the UAE showed satisfaction of the participating patients with the quality of the telehealth experience. The feedback from the patients indicated that telehealth services are patient-centered, and hence, they are responsive to individual patient preferences, needs, and values. However, patients declared some challenges such as technical issues while trying to connect to the telehealth consultation online, the high cost, and insurance coverage [49].

Though telehealth has the advantage of extending access to care, especially to remote and rural areas, Schwamm [50] emphasized that low socioeconomic status and digital literacy are major barriers to telehealth equitability.

Furthermore, El Hayek et al. [51] pointed to the unavailability of telepsychiatric guidelines in the Arab Gulf countries during the pandemic and reported some cultural barriers to implementing telepsychiatry in these countries including religion, gender sensitivity, and other societal norms. Reports from other countries including Singapore, the UK, and the USA indicated the establishment of guidelines for both clinicians and patients on using telepsychiatry during the pandemic [52].

Therapeutic Approaches

Exposure and response prevention and pharmacotherapy remain the treatment of choice, even though the setting in which treatment is conducted has been shifted. Changes in the assessment and treatment approaches of OCD have occurred to personalize care and be aligned with public health guidelines. Treatment personalization should still be made to ensure safety for both patients and providers while balancing efficacy and patient preferences [53].

Therapists during the pandemic changed their therapeutic approaches and they focused on changing points of reference, providing practical advice on coping for OCD patients and their families, stimulating them to engage in exposure, and encouraging patients to seek social support. Furthermore, they involved family members in therapy to be validating, supportive, and encouraging, without accommodating the OCD behavior [54].

Another aspect of OCD management has been shown during the pandemic. Some studies have shown the positive effects of physical activity for those with depression and OCD and highlighted the importance of being physically active as a measure of resilience [55].

Discussion

Synthesis of Findings

According to the available studies from the Gulf countries, it seems that the COVID-19 crisis was associated with an increased prevalence of OCD in the seven countries despite the unique dynamics of COVID-19 for each country. However, all the existing studies are cross-sectional and have used convenience samples. Such studies cannot establish a causation relationship between the pandemic and OCD or determine the magnitude of change in the prevalence of this psychiatric morbidity during the pandemic in comparison to the pre-pandemic situation. However, these studies identified some sociocultural factors associated with the appearance of new OCD symptoms or the worsening of diagnosed ones, which may help in the prevention and early intervention of future pandemics. Studies also revealed that people with pre-existing psychological disorders or OCD in particular were more vulnerable to the negative impact of the pandemic and emphasized that they need special care.

Comparing the current results is challenging due to the different settings, timing, and different OCD assessment tools. Studies that were carried out early in the pandemic (July to November 2020) when the public suffered widespread lockdowns, a high disease burden, and much uncertainty might have overestimated the OCD symptoms.

Globally, several studies in Western countries and China have investigated the psychological aspects of the COVID-19 pandemic and exposed both the development of new symptoms and the worsening of the already existing symptoms of OCD during the COVID-19 pandemic [56,57]. Tanir et al. [56] also concluded that OCD symptoms may worsen due to additional emphasis on self-hygiene in the current health advice for preventing COVID-19 contamination. Moreover, Davide et al. [58] highlighted a significant increase in the severity of obsession and compulsion in adults with OCD.

The observed negative impact of the COVID-19 pandemic on OCD symptoms in the Arabian Gulf countries goes in hand with the current evidence built from systematic reviews of studies from various countries. A systematic review of studies that were conducted in the Spring/Summer of 2020 showed that the COVID-19 pandemic was a strong stressor for the development of new OCD symptoms in the general population as well as the worsening of symptoms in previously diagnosed individuals [59]. Another recent review of studies completed in 2020 and 2021 also showed a significant impact of the COVID-19 pandemic on OCD symptoms mainly in the form of worsening of OCD symptoms, especially contamination and washing [60]. Furthermore, Linde et al. [61] in their systematic review confirmed increased OCD symptoms to a clinically significant degree in 40% of OCD patients [61].

Implications for Practice

The findings of the reviewed studies indicate that public health officials must increase their focus on raising awareness of the general public about mental health during a pandemic. Increasing the awareness will help in limiting the stigma around this issue. Close monitoring of patients with OCD is essential to prevent the impairment of symptoms and the development of new cases. Planning multidisciplinary strategies to enhance access to mental health services during a pandemic is essential.

Several groups are particularly more vulnerable to depression, anxiety, obsessions, and compulsions, and are most affected by the pandemic and lockdowns. Therefore, policymakers should prioritize their attention to them to help them make informed adjustments to restrictive measures. The presence of facilitating plans for daily physical activity during lockdowns is a critical measure to counteract the negative impact of a pandemic. The health and education authorities should provide more support to schools, universities, sports organizations, and mental health organizations to minimize the negative impacts of lockdowns. Furthermore, authorities should consider allocating more resources to support those lonely people, like single and divorced individuals. For example, supportive text messages can effectively reduce anxiety and depressive symptoms.

Limitations

The reviewed studies are limited by adopting the cross-sectional design which cannot imply causation. The online survey was the tool used to collect data and this is limited by some bias as only participants with access to social media platforms were able to participate, and the questionnaire was self-administered, which may have caused recall bias. However, well-validated instruments have been used, which may minimize these biases. The self-reported responses do not provide an actual diagnosis of OCD, they only offer an initial assessment, and no clinical interviews were followed to confirm the diagnosis.

Future Research Directions

The precise relationship between the COVID-19 pandemic and the onset of OCD remains unclear. Longitudinal studies are necessary to observe the gradual change in OCD symptoms, diagnosis, or severity in response to changing restrictive measures. Future studies should include clinical interviews to confirm the OCD diagnosis and focus on the factors affecting the emergence or severity of OCD symptoms.

## Conclusions

A review of the literature reveals a paucity of local studies and reports from the Arab Gulf region that focus on the impact of the novel COVID-19 on OCD. Most studies originate from Saudi Arabia and the United Arab Emirates, with a few originating from Kuwait, Qatar, Oman, and Bahrain. The global pandemic of COVID-19 has had a deleterious impact on the prevalence of OCD in Arab Gulf countries. The crisis has been associated with an increased prevalence of OCD, new onset of symptoms, and worsening and exacerbations of existing manifestations. The observed effects were nearly similar across all seven Gulf countries. Studies conducted during the initial stages of the pandemic showed a much higher prevalence than those done after the pandemic, at the end of the year 2021. 
